# High Production of 2,3-Butanediol (2,3-BD) by *Raoultella ornithinolytica* B6 via Optimizing Fermentation Conditions and Overexpressing 2,3-BD Synthesis Genes

**DOI:** 10.1371/journal.pone.0165076

**Published:** 2016-10-19

**Authors:** Taeyeon Kim, Sukhyeong Cho, Sun-Mi Lee, Han Min Woo, Jinwon Lee, Youngsoon Um, Jin-Ho Seo

**Affiliations:** 1 Interdisciplinary program in agriculture biotechnology, College of Agriculture and Life Science, Seoul National University, 1 Gwanak-ro, Gwanak-gu, Seoul, 151–742, Republic of Korea; 2 Clean Energy Center, Korea Institute of Science and Technology (KIST), Seongbuk-gu, Seoul, 136–791, Republic of Korea; 3 Clean Energy and Chemical Engineering, Korea University of Science and Technology, Daejeon, Republic of Korea; 4 Department of Agricultural Biotechnology and Center for Food and Bioconvergence, Seoul National University, Seoul, 151–742, Republic of Korea; 5 Department of Food Science and Biotechnology, Sungkyunkwan University (SKKU), 2066 Seobu-ro, Jangan-gu, Suwon, 16419, South Korea; 6 Korea C1 gas refinery R&D center, Sogang University, Seoul, 121–742, Republic of Korea; 7 Department of Chemical and Biomolecular Engineering, Sogang University, Seoul, 121–742, Republic of Korea; National Renewable Energy Laboratory, UNITED STATES

## Abstract

Biological production of 2,3-butandiol (2,3-BD) has received great attention as an alternative to the petroleum-based 2,3-BD production. In this study, a high production of 2,3-BD in fed-batch fermentation was investigated with a newly isolated bacterium designated as *Raoultella ornithinolytica* B6. The isolate produced 2,3-BD as the main product using hexoses (glucose, galactose, and fructose), pentose (xylose) and disaccharide (sucrose). The effects of temperature, pH-control schemes, and agitation speeds on 2,3-BD production were explored to optimize the fermentation conditions. Notably, cell growth and 2,3-BD production by *R*. *ornithinolytica* B6 were higher at 25°C than at 30°C. When three pH control schemes (no pH control, pH control at 7, and pH control at 5.5 after the pH was decreased to 5.5 during fermentation) were tested, the best 2,3-BD titer and productivity along with reduced by-product formation were achieved with pH control at 5.5. Among different agitation speeds (300, 400, and 500 rpm), the optimum agitation speed was 400 rpm with 2,3-BD titer of 68.27 g/L, but acetic acid was accumulated up to 23.32 g/L. Further enhancement of the 2,3-BD titer (112.19 g/L), yield (0.38 g/g), and productivity (1.35 g/L/h) as well as a significant reduction of acetic acid accumulation (9.71 g/L) was achieved by the overexpression of homologous *budABC* genes, the 2,3-BD-synthesis genes involved in the conversion of pyruvate to 2,3-BD. This is the first report presenting a high 2,3-BD production by *R*.*ornithinolytica* which has attracted little attention with respect to 2,3-BD production, extending the microbial spectrum of 2,3-BD producers.

## Introduction

2,3-butanediol (2,3-BD) is a promising platform material with a wide range of industrial applications as a solvent and a precursor for methyl ethyl ketone, 1,3-butanedien, and 2-butene [1,2]. Those 2,3-BD-derived materials can be also used as monomers for synthetic rubber and other polymers [3,4]. Instead of petroleum-based 2,3-BD production, biological production of 2,3-BD has received much attention as an environmentally friendly alternative method. There are substantial studies on 2,3-BD production by microbial fermentation using carbohydrates through the optimization of fermentation conditions and genetic engineering. Ma et al. [5] reported a high production of 2,3-BD (150 g/L) with *Klebsiella pneumoniae* SDM through optimization of feeding strategy. *Serratia marcescens* H30 (a random mutant of *S*. *marcescens* A3) and a surfactant-deficient mutant of *S*. *marcescens* H30 produced 139.92 g/L and 152 g/L 2,3-BD by optimization of the medium composition [6] and inactivation of the *swrW* gene [7], respectively. The production of 2,3-BD by *Enterobacter aerogenes*, *K*. *pneumoniae* and *K*. *oxytoca* was reported to be dramatically increased by deletion of NADH-requiring by-product synthesis genes [8–11] and the overexpression of 2,3-BD synthesis related genes [12–14]. In addition to those microorganisms, well-known model hosts such as *Saccharomyces cerevisiae* [3] and *Escherichia coli* [15] have also been engineered to produce 2,3-BD.

Among the 2,3-BD producers, *Klebsiella* species, such as *K*. *pneumoniae* [5] and *K*. *oxytoca* [13], have shown superior 2,3-BD production. Other *Klebsiella* species have not been extensively investigated as 2,3-BD producers except for studies on the characterization of the 2,3-BD synthesis genes in *K*. *terrigena* by Blomqvist et al. [16] and Mayer et al. [17]. Meanwhile, several *Klebsiella* species including *K*. *terrigena*, *K*. *ornithinolytica*, *and K*. *planticola* have been reclassified to the *Raoultella* genus [18]. *Raoultella* species (*R*. *terrigena*, *R*. *planticola and R*. *ornithinolytica*) are Gram-negative, non-motile, aerobic and facultative anaerobic having a respiratory as well as a fermentative metabolism [18]. Unlike *Klebsiella* species which have important human medical issues, *Raoultella* species are generally isolated from environmental samples, insects, and fishes [18,19]. *R*. *terrigena* has been investigated for the production of pullulanase, a debranching enzyme hydrolyzing pullulan and branched polysaccharides [20]. Polysaccharide-protein complex and Tris-peptide complex from *R*. *ornithinolytica* have been shown to have antifungal-anticancer effects [21] and antitumour-apoptotic effects [22], respectively. However, the use of *Raoultella* species for biological production of chemicals through fermentation as well as metabolic engineering has not been reported to date.

In this study, we investigated a newly isolated bacterium designated as *R*. *ornithinolytica* B6 as another promising candidate microbe for the high production of 2,3-BD. *R*. *ornithinolytica* B6 produced 2,3-BD as the main product using hexose, pentose, and sucrose. The optimized fermentation conditions of *R*. *ornithinolytica* B6 were established by comparing 2,3-BD production at different temperatures, pH control schemes, and agitation speeds. To further increase the carbon flux toward 2,3-BD synthesis and to decrease by-product formation, genetic engineering was conducted by overexpressing 2,3-BD synthesis related genes: *budB*, *budA*, and *budC* encoding acetolactate synthase, acetolactate decarboxylase, and acetoin reductase (AR), respectively. The results validated that *R*. *ornithinolytica* B6 has potential as a high 2,3-BD producer. This is the first study on optimization of fermentation conditions and genetic modification for 2,3-BD production with *R*. *ornithinolytica*.

## Materials and Methods

### Media

The enrichment medium contained 0.5 g/L K_2_HPO_4_, 0.5 g/L KH_2_PO_4_, 3 g/L (NH_4_)_2_SO_4_, 0.2 g/L MgSO_4_∙7 H_2_O, 0.02 g/L CaCl_2_∙2 H_2_O, 0.0092 g/L FeSO_4_∙7 H_2_O, 1 g/L yeast extract, 30 g/L glycerol, 0.0108 g/L calcium pantothenate, 0.0108 g/L nicotinic acid, 0.2688 g/L myo-inositol, 0.0108 g/L thiamine, 0.0108 g/L pyridoxine, 0.0022 g/L para-aminobenzoic acid, 0.00003 g/L d-biotin, 1 mL of SL7 solution containing 0.025% HCl, 70 mg/L ZnCl_2_, 100 mg/L MnCl_2_∙4H_2_O, 60 mg/L H_3_BO_3_, 200 mg/L CoCl_2_∙6 H_2_O, 20 mg/L CuCl_2_∙2 H_2_O, 20 mg/L NiCl_2_∙6 H_2_O, and 40 mg/L NaMoO_4_∙2 H_2_O. The initial pH of the medium was adjusted to 6.5 with 5 N KOH. The fermentation medium [23] contained 13.7 g/L K_2_HPO_4_, 2 g/L KH_2_PO_4_, 3.3 g/L (NH_4_)_2_HPO_4_, 6.6 g/L (NH_4_)_2_SO_4_, 0.25 g/L MgSO_4_∙7 H_2_O, 0.05 g/L FeSO_4_∙7 H_2_O, 0.001 g/L ZnSO_4_∙7 H_2_O, 0.001 g/L MnSO_4_∙H_2_O, 0.01g/L CaCl_2_∙2 H_2_O, and 50 g/L sugar. 5 g/L yeast extract and 10 g/L casamino acid were added as indicated in the text.

### Isolation and Identification

A 2,3-BD-producing bacterium was isolated from Baegun Mountain in Korea. There is no requirement of specific permission for soil samples collection. The sampling site is public area in urban mountain without access restriction. The sampling did not involve endangered species or protected habitats. The soil sample was cultivated in enrichment medium at 30°C. After enrichment, samples were grown on agar plates and single isolated colonies were transferred to 20 ml of the enrichment medium. These procedures were repeated several times to ensure the purity of the culture. The 2,3-BD producing bacterium was selected by analysis of metabolites with gas-chromatography.

For the bacterium identification, genomic DNA (gDNA) was extracted using Exgene™ Cell SV mini kit (GeneAll Biotechnology Co.,LTD, Korea). The extracted gDNA was used as template and universal primers 27 F and 1492 R were used for the amplification of 16S rRNA gene. The sequences of PCR product (1354 bp) was analyzed by Macrogen Inc. (Seoul, Korea) (http://www.macrogen.com). Sequences from the nearest relatives were identified with the Ez-taxon program [24]. The isolate was deposited as KCCM11176-P in the Korea Culture Center of Microorganisms. Recently, the full genome sequence of *R*. *ornithinolytica* B6 was analyzed and reported by Marcrogen Inc. [25]. The complete genome sequence of *R*. *ornithinolytica* B6 has been deposited at GenBank under accession number CP004142.

### Flask culture

A single colony from an agar plate was inoculated in LB medium and incubated on a shaking incubator at 150 rpm overnight at 30°C or 25°C. Then, the seed culture was inoculated with 2% (v/v) in 20 ml of fermentation medium with 50 g/L glucose. Sampling was carried out periodically to measure pH, cell growth, residual substrates, and products. The data were presented as average ± standard deviation of triplicate experiments.

### Fed-batch fermentation

To optimize fermentation conditions for 2,3-BD production by *R*. *ornithinolytica* B6, fed-batch fermentation was performed under various pH conditions and agitation speeds. At first, the experiments were conducted to find the optimum pH for fed-batch fermentation. A fed-batch culture was carried out in a 3-L bioreactor (Fermentec, Korea) with 1 L of the initial medium. The seed culture (10% v/v) was inoculated into the fermentation medium with an initial pH 7.0. The cultivation was carried out at 25°C and 200 rpm for the agitation speed with a 0.5 vvm air flow in a defined medium, and pH was controlled as follows: (i) without pH control, (ii) maintaining the initial pH, (iii) maintained at pH 5.5 after naturally decreasing to pH 5.5. To control pH, 5 N KOH was added automatically. The agitation speed effect was investigated under the following conditions: 25°C, air flow at 1 vvm, and stirring at 300, 400 and 500 rpm. When the residual glucose concentration dropped below 20 g/L, 100 mL of glucose stock solution (600 g/L) was supplied.

### Acetoin reductase (AR) activity assay

To estimate the AR activity toward acetoin reduction and 2,3-BD oxidation, a *budC* (encoding AR) overexpression mutant was constructed. The plasmids pUC18CM and pUC18CM-*budC* were constructed as previously reported [1]. The chloramphenicol resistant gene was amplified from the 708-FLPe plasmid (Gene Bridges, Germany). The 829 bp *Aat*II-*Aat*II fragment of the chloramphenicol resistant gene was ligated into pUC18 (GentScript, USA) for the construction of pUC18CM. The primers for the overexpression of *budC* were budC-F (5’-TTTTCTAGAATGCAAAAAGTCGCC-3’) (*Xba*I site underlined) and budC-R (5’-TTTAAGCTTTTAGTTAAAAACCATACCG-3’) (*Hind*III site underlined). The PCR product was inserted into the *Xba*I and *Hind*III restriction sites of pUC18CM. *E*. *coli* HIT-DH5α (RBC Bioscience Corp, Taiwan) was used to clone these genes. The transformation of pUC18CM-*budC* to *R*. *ornithinolytica* B6 was done by electroporation of the plasmid at 12.5 kV/cm, 200 Ω, and 25 μF with Gene PulserXcell (Bio-rad).

*R*. *ornithinolytica* B6 (pUC18CM-*budC*) was cultivated in 20 mL LB medium with 30 μg/mL of chloramphenicol for 12 hours at 25°C. A harvested cell pellet was suspended in 50 mM potassium phosphate buffer (pH 7.0) and sonicated with the VCX 750 (SONICS & MATERIALS, INC., USA). Before measurement of the AR activity, the buffer was exchanged with a fresh buffer using an Amicon ultrafiltration unit with a 10 kDa cut-off (Millipore, Billerica, MA, USA), and crude proteins larger than 10 kDa including AR (27 kDa) were used for the AR activity assay. The activities of AR for acetoin reductase and 2,3-BD oxidation were assayed as previously described [13]. Briefly, acetoin reduction activity of AR was determined spectrophotometrically by measuring the consumed NADH at 340 nm using the molar extinction coefficient of NADH over 5 minutes (Cary 60 UV-Vis, Agilent Technologies, USA). The reaction mixtures containing 50 mM potassium phosphate buffers (pH 7) with 0.15 mM NADH and 1.0 mM acetoin were incubated for 5 minutes at 25°C. After adding crude protein (25 ug/mL) the reaction was started. One unit of AR activity was defined as the amount of crude protein required to reduce 1 μmol of NADH in 1 min. The 2,3-BD oxidation activity assay was at 25°C and pH 7.0 with 0.15 mM NAD^+^ and 1.0 mM 2,3-BD. 2,3-BD oxidation activity of AR was analyzed by measuring the generated NADH in absorbance at 340 nm.

### Overexpression of *budABC*

The genes encoding acetolactate decarboxylase (*budA*) (accession number AGJ85611), acetolactate synthase (*budB*) (accession number AGJ85610), and AR (*budC*) (accession number AGJ85609) were derived from the full genome of *R*. *ornithinolytica* B6. The *budA*, *budB* and *budC* genes consist of the *bud* operon with an amino acid similarity of 96%, 95% and 96% to the corresponding genes of *K*. *terrigena* [16]. The primers for the overexpression of *budABC* were budA-F (5’-TTTGAATTCCACAAGACTAAGGAGGCCA CAATGACCCATTCTTCTGC-3’) (*EcoR*I site underlined) and budC-R (5’-TTTCTCGAGTTAGTTAAAAACCAT ACCG-3’) (*Xho*I site underlined). The PCR product was inserted into the *EcoR*I and *Xho*I restriction sites of pBbA5c-RFP (Addgene, USA) [26], resulted in pBbA5c-*budABC*. The pBbA5c-RFP vector contains p15A origin, IPTG inducible gene expression system with lac promoter, and chloramphenicol resistance marker. The pBbA5c-RFP contains the restriction enzyme site of, *EcoR*I, *Bgl*II, *BamH*I, and *Xho*I, in sequence. The *rfp* gene is located between the restriction site of *Bgl*II and *BamH*I. Because the cleavage with *Bgl*II and *BamH*I produces compatible overhangs, the empty vector, pBbA5c, was constructed through the digestion of pBbA5c-RFP with *Bgl*II and *BamH*I followed by the ligation. Target genes were induced with 0.5 mM IPTG after 3 hours of incubation. MAX Efficiency^®^ DH10B™ (Invitrogen, USA) was used to clone these genes. The genotype of the competent cells was F^-^*mcr*A Δ(*mrr*-*hsd*RMS-*mcr*BC) φ80*lac*ZΔM15 Δ*lac*X74 *rec*A1 *end*A1 *ara*D139 Δ(*ara*, *leu*)7697 *gal*U *gal*K λ^-^*rps*L *nup*G/pMON14272/pMON7124. *R*. *ornithinolytica* B6 was used as a host strain, and transformation was done by electroporation as described above. *R*. *ornithinolytica* B6 harboring pBbA5c-*budABC* and pBbA5c was cultured with 30 μg/mL of chloramphenicol to maintain the plasmid.

### SDS-PAGE for confirmation of *budABC* overexpression

*R*. *ornithinolytica* B6, *R*. *ornithinolytica* B6 (pBbA5c), *R*. *ornithinolytica* B6 (pBbA5c-RFP), and *R*. *ornithinolytica* B6 (pBbA5c-*budABC*) were grown in the medium described in the materials and method section. Except the wild type, 30 μg/mL of chloramphenicol was added. When OD_600_ was reached at 1.0, 0.5 mM IPTG was added. After 3 hours of induction, the cells were harvested to obtain soluble enzymes using BugBuster Master Mix (Merch, Germany). The enzyme of 10 ug was loaded onto the 10% SDS-PAGE gel. The electrophoresis was performed using 1/10 diluted 10x Tris/Glycine/SDS buffer (Bio-rad, USA) at 200 V for 40 min. The gel was stained using Coomassie Brilliant Blue R250 staining solution (Bio-rad, USA).

### Analytical procedures

Cell growth was measured by the optical density of the culture broth at 600 nm with a spectrophotometer (Shimadzu UV-1240, Japan). The concentrations of sugars and organic acids were analyzed using high performance liquid chromatography (Agilent HPLC, USA) equipped with an Aminex HPX-87 H column [300×7.8 mm] (Bio-rad, USA). The operation conditions of HPLC were as follows: column temperature, 50°C; flow rate of the mobile phase (5 mM H_2_SO_4_), 0.6 mL/min, and UV detector wave length, 210 nm. The quantifications of the fermentation products such as acetoin, 2,3-BD, and ethanol were determined using gas chromatography (Shimadzu GC-2010, Japan) equipped with a flame ionization detector and HP-INNOWAX column (30 m×0.32 mm, 0.25 μm) (Agilent, USA) under the following conditions: oven temperature, from 50°C to 240°C at a rate of 30°C/min; injector temperature, 240°C; detector temperature, 250°C, and carrier gas (N_2_) flow rate, 30 mL/min.

## Results

### Identification of the 2,3-butanediol-producing isolate

**Fig 1:**
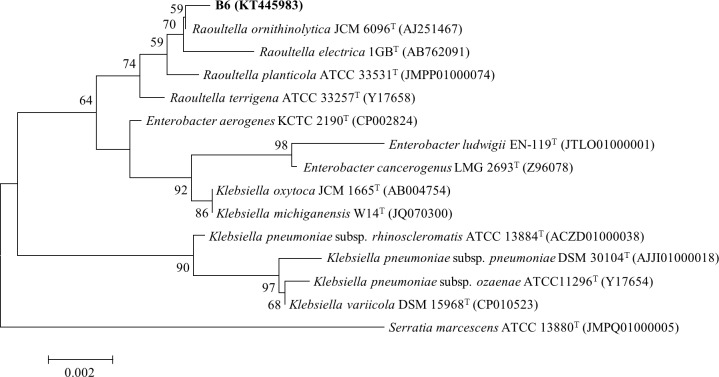
Phylogenetic tree derived from the analysis of the 16S rRNA gene sequences of isolate B6 and related strains. The tree was inferred using the Neighbor-Joining method. The evolutionary distances were computed using the Maximum Composite Likelihood method. Bootstrap values are from 1000 replications and only those greater than 50% are shown. *Serratia marcescens* ATCC 13880 was selected as the out group. Evolutionary analyses were conducted in MEGA7. The scale bar indicates 0.2% nucleotide substitution rate.

The microorganism isolated from the Baegun Mountain soil sample was identified with 16s rDNA gene sequences. According to the phylogenetic analysis with the 16S rDNA gene sequences, the isolate was affiliated to the genus *Raoultella* with the highest 16S rDNA sequence similarity of 99.9% to *R*. *ornithinolytica* JCM6096^T^ (Fig 1). The isolate was designated as *R*. *ornithinolytica* B6 and deposited in the Korea Culture Center of Microorganisms (KCCM) as KTCM11176-P.

### Effect of temperature on *R*. *ornithinolytica* B6 fermentation

*R*. *ornithinolytica* (formerly known as *K*. *ornithinolytica*) is one of the *Raoultella* species identified from environmental samples, and it can grow at relatively low temperatures, even at 10°C [18]. To evaluate the effect of temperature on cell growth and 2,3-BD production, flask cultivation was conducted at 25°C, 30°C, and 37°C with 50 g/L of glucose as a carbon source. As shown in Table 1, cell growth, 2,3-BD concentration, and productivity were higher at 25°C than at 30°C by 58.7%, 5.1%, and 4.5%, respectively. Moreover, the total organic acid production was much lower at 25°C compared to that at 30°C (1.13 vs. 3.34 g/L) (Table 1). Surprisingly, at 37°C, cell growth was not observed during 48 hours of cultivation (data not shown). This phenomenon is distinguished from other 2,3-BD producing microorganisms such as *K*. *pneumoniae* and *K*. *oxytoca* cultivated at 37°C for 2,3-BD production [5,8,10]. Because the cultivation of *R*. *ornithinolytica* B6 at 25°C appeared to be effective for cell growth and 2,3-BD production, all further studies were done at 25°C.

**Table 1:** Effect of culture temperature on 2,3-BD production performance by *R*. *ornithinolytica* (initial glucose at 50 g/L and pH 6.5).

	Time (hours)	pH	OD at 600nm	2,3-BD (g/L)	Acetoin (g/L)	Ethanol (g/L)	Total organic acid^1)^ (g/L)	2,3-BD yield (g/g)	2,3-BD productivity (g/L/h)
25°C	36	5.36 ± 0.01	11.49 ± 0.26	14.68 ± 0.34	2.48 ± 1.03	3.17 ± 0.31	1.13 ± 0.75	0.29 ± 0.01	0.41 ± 0.01
30°C	36	4.97 ± 0.26	7.24 ± 0.05	14.05 ± 0.20	2.13 ± 0.26	3.02 ± 0.26	3.34 ± 0.79	0.30 ± 0.02	0.39 ± 0.01

^1)^ Organic acids including acetic acid, succinic acid, and lactic acid

The data are given as average ± standard deviation of triplicate experiments

### Carbon source utilization by *R*. *ornithinolytica* B6

Because lignocellulosic biomass [27] and marine biomass [28] are composed of various sugars and these materials have received attention as carbon sources of microorganisms for producing value-added materials, utilization of various carbon sources is an essential feature of industrial microorganisms. Thus, various sugar substrates (glucose, galactose, fructose, xylose and sucrose) were used to evaluate the carbon source utilization capability of *R*. *ornithinolytica* B6. All the tested substrates supported the growth of *R*. *ornithinolytica* B6, and 2,3-BD was produced as a main product with varying levels of by-products (Table 2).

**Table 2:** Production of 2,3-BD and other metabolites using various carbon sources at 25°C (initial carbon source at 50 g/L and pH 7.0).

Carbon sources	Time (hours)	pH	OD at 600nm	2,3-BD (g/L)	Acetoin (g/L)	Ethanol (g/L)	Total organic acid ^1)^ (g/L)	2,3-BD yield (g/g)	2,3-BD productivity (g/L/h)
Glucose	36	5.71 ± 0.00	10.72 ± 0.45	21.28 ± 1.16	1.03 ± 0.26	3.30 ± 0.02	0	0.34 ± 0.01	0.59 ± 0.03
Fructose	36	5.59 ± 0.01	10.43 ± 0.86	20.64 ± 1.70	1.04 ± 0.22	3.01 ± 0.01	0.39 ± 0.55	0.31 ± 0.01	0.57 ± 0.05
Galactose	36	5.72 ± 0.03	11.28 ± 0.37	19.68 ± 1.94	1.02 ± 0.12	3.52 ± 0.16	1.10 ± 0.03	0.24 ± 0.03	0.55 ± 0.05
Xylose	48	5.03 ± 0.06	13.61 ± 0.34	18.75 ± 1.91	1.56 ± 0.07	2.52 ± 0.01	3.65 ± 0.02	0.24 ± 0.02	0.39 ± 0.02
Sucrose	24	5.94 ± 0.02	13.94 ± 0.09	16.35 ± 0.37	5.66 ± 0.10	2.38 ± 0.09	4.07 ± 0.92	0.29 ± 0.01	0.68 ± 0.02

^1)^ Organic acids including acetic acid, succinic acid, and lactic acid

The data are given as average ± standard deviation of triplicate experiments

When glucose was supplied, the best 2,3-BD production performance was achieved with the titer of 21.28 g/L and the yield of 0.34 g/g. Thus, glucose was chosen as the sole carbon source for further experiments.

### Effect of pH-control on 2,3-BD production during fed-batch fermentation

**Fig 2:**
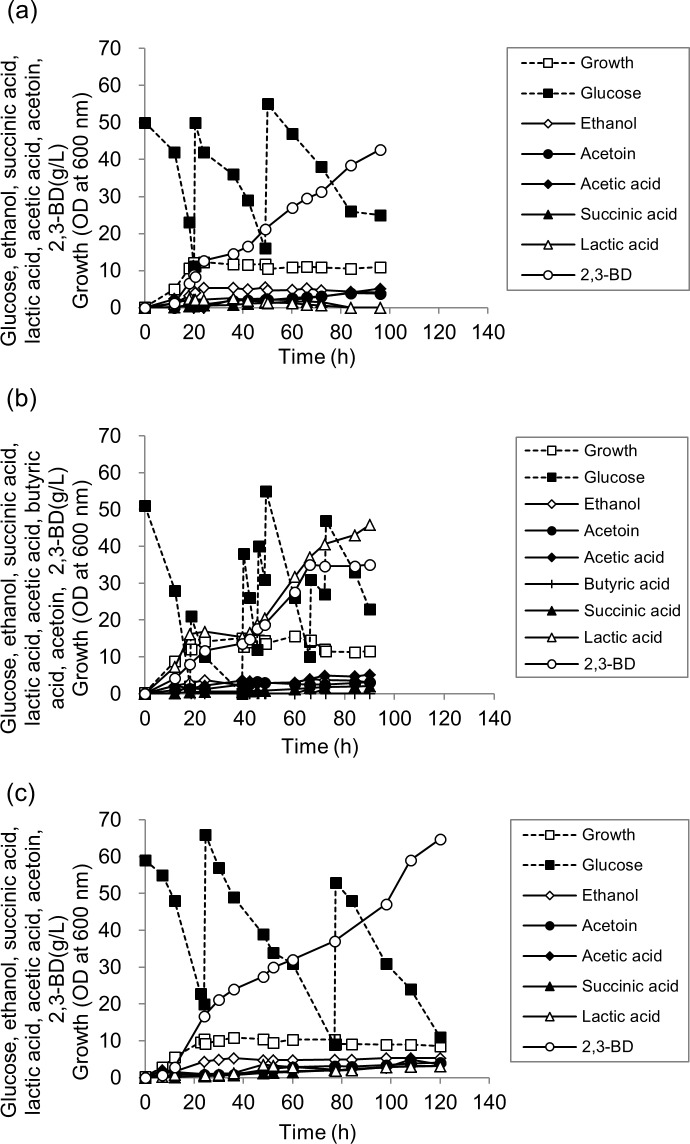
**Fed-batch fermentation of glucose by *R*. *ornithinolytica* B6 with an agitation speed of 200 rpm;** (a) without pH control; (b) with pH maintained at 7.0; (c), pH controlled at 5.5 after pH dropped to 5.5 from the initial pH of 7.0.; ■, glucose; □, growth; ♢, ethanol; ●, acetoin; ○, 2,3-BD; ♦, acetic acid; ▲, succinic acid; △, lactic acid; +, butyric acid.

In the batch cultures of *R*. *ornithinolytica* B6, acid formation along with 2,3-BD production generally occurred causing a pH drop. To investigate the effect of pH on 2,3-BD production and acid formation in fed-batch fermentation, three pH-control schemes with the initial pH at 7 were tested: i) no pH control; ii) pH control at 7, and iii) pH control at 5.5 once the pH decreased to 5.5 during fermentation. When the pH was not controlled, the pH decreased from pH 7.0 to pH 5.1 during 42 hours of fermentation and further decreased to 4.6 after 96 hours. The consumption of glucose was 136.00 g/L, and 42.67 g/L of 2,3-BD was produced after 96 hours (Fig 2A).

In the case of pH control at 7, the cell growth was similar to that of no pH control; but, the glucose consumption was higher than that of the no pH control by 1.4-fold (196.89 g/L). Interestingly, the main product was lactic acid (45.84 g/L) not 2,3-BD (35.01 g/L) probably because of a high pH set point. When the pH was adjusted to 5.5 after 36 hours, *R*. *ornithinolytica* B6 consumed 202.60 g/L of glucose and produced 64.74 g/L of 2,3-BD as the main product after 120 hours (Fig 2C). Moreover, adjusting the pH to 5.5 effectively reduced lactic acid formation (3.12 g/L) compared to the fed-batch fermentation at pH 7.

Considering the previous results by Jung et al. [8] and Park et al. [9] in which the enhancement of 2,3-BD production was obtained by reducing lactic acid formation with lactate dehydrogenase-deleted mutants, the two stage pH control strategy (controlling pH at 5.5 after the pH reached 5.5) attempted here would be a relatively simple and convenient method to reduce lactic acid production. Because the 2,3-BD production (64.74 g/L), yield (0.32 g/g), and productivity (0.54 g/L/h) obtained with pH control at 5.5 were higher than those of the other two pH scheme fed-batch fermentations, all further studies were done by maintaining the pH at 5.5 after the pH dropped from 7 to 5.5.

### Effect of agitation speed on 2,3-BD production in fed-batch fermentation

The oxygen supply strategy by adjusting the agitation speed during fermentation is a critical factor for a high production of 2,3-BD [2,9,13]. The effect of the agitation speed on 2,3-BD production by *R*. *ornithinolytica* B6 was investigated at agitation speeds of 300 rpm, 400 rpm, and 500 rpm in fed-batch fermentation (Table 3).

**Table 3:** Comparison of products in fed-batch fermentation from *R*. *ornithinolytica* B6, *R*. *ornithinolytica* B6 (pBbA5c), and *R*. *ornithinolytica* B6 (pBbA5c-*budABC*) with the agitation speed of 400 rpm.

	*R*. *ornithinolytica* B6			*R*. *ornithinolytica* B6 (pBbA5c)	*R*. *ornithinolytica* B6 (pBbA5c-*budABC*)
Agitation speed (rpm)	300	400	500	400	400
Fermentation time (h)	95	87.5	105	85	83
Consumed substrate (g/L)	203.44	227.29	91.52	194.64	294.40
O.D. at 600 nm	16.35 [18.84]^1)^	23.33 [33.70]	32.50 [37.49]	30.74 [36.27]	39.17 [41.14]
2,3-BD (g/L)	54.21	68.27	7.90	61.12	112.19
Acetoin (g/L)	12.05 [12.91]	8.80 [11.26]	1.60 [1.73]	7.82 [11.32]	22.02 [23.55]
Acetic acid (g/L)	14.70	23.32	18.10	23.23	9.71 [9.78]
Lactic acid (g/L)	2.19 [8.40]	1.20 [3.53]	0.00	0.34 [1.59]	0.73 [1.52]
Succinic acid (g/L)	4.17 [7.74]	0.00 [5.09]	0.00	0.00 [2.47]	0.00 [7.42]
Ethanol (g/L)	5.16 [8.65]	1.21 [6.29]	0.00	1.25 [6.04]	2.68 [6.25]
2,3-BD yield (g/g)	0.27	0.30	0.08	0.31	0.38
2,3-BD productivity (g/L/h)	0.57	0.78	0.07	0.72	1.35

^1)^ Values in [] represent the maximum value for each measurement during the fermentation.

**Fig 3:**
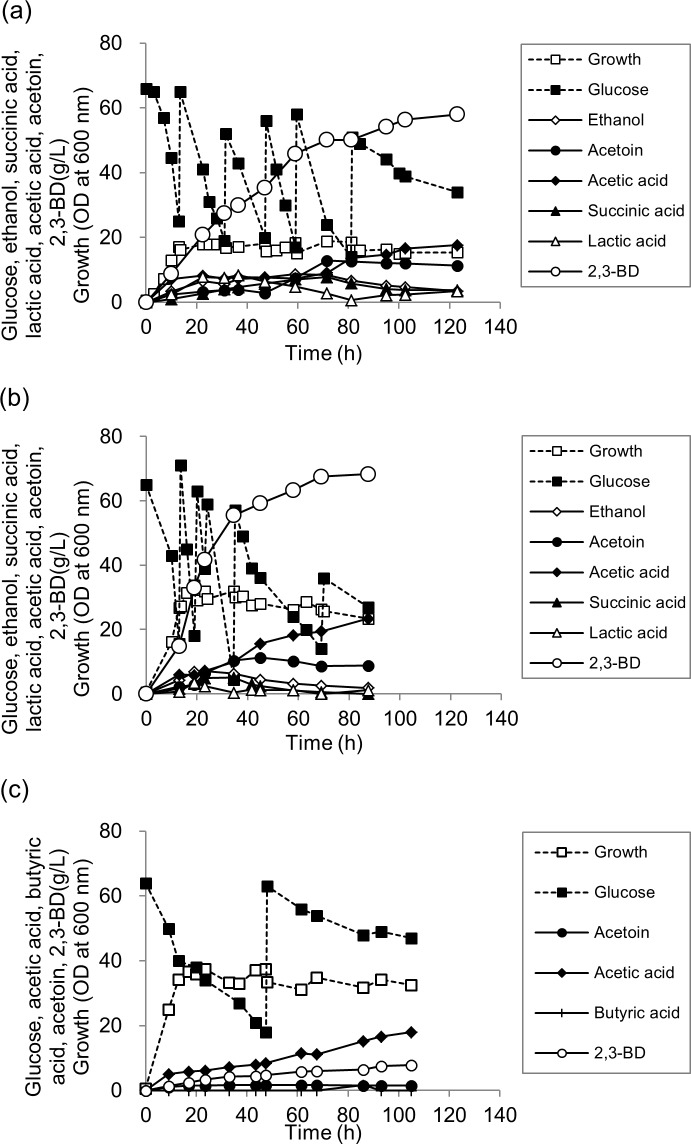
Fed-batch fermentation of glucose by *R*. *ornithinolytica* B6 at various agitation speeds. pH controlled at 5.5 after the pH reached 5.5 from an initial pH of 7.0 under several agitation speeds (a) 300 rpm, (b) 400 rpm, (c) 500 rpm; ■, glucose; □, growth; ♢, ethanol; ●, acetoin; ○, 2,3-BD; ♦, acetic acid; ▲, succinic acid; △, lactic acid; +, butyric acid.

To enhance cell growth and 2,3-BD production, 5 g/L yeast extract and 10 g/L casamino acid were added to the medium as previously described [8,13]. When the agitation speed was controlled at 300 rpm, 2,3-BD was produced up to 54.21 g/L with the productivity of 0.57 g/L/h. Acetoin and acetic acid were also produced up to 12.05 g/L and 14.70 g/L, respectively (Fig 3A).

When the agitation speed of 400 rpm was tested, 2,3-BD production was enhanced up to 68.27 g/L with the productivity of 0.78 g/L/h and the yield of 0.30 g/g (Fig 3B). Interestingly, at agitation speeds of 300 rpm and 400 rpm, the formation of NADH-requiring by-products such as lactic acid, succinic acid, and ethanol reached the maximum point during the fermentation and then decreased (Table 3). The maximum as well as the final concentrations of those by-products were lower at 400 rpm than at 300 rpm, indicating that 400 rpm was efficient to reduce by-product formation. In contrast, acetic acid, a none NADH-requiring by-product, continuously increased during the fermentation, and acetic acid concentration at 400 rpm was higher than that at 300 rpm by 1.59 fold. In addition, cell growth was much enhanced at 400 rpm compared to that at 300 rpm. At the agitation speed of 500 rpm, cell growth was further enhanced, and acetic acid (18.10 g/L) was even the main product with the yield of 0.19 g/g. Production of 2,3-BD was only 7.90 g/L during 105 hours of fermentation (Fig 3C).

These results indicate that a high agitation speed stimulated acetic acid synthesis and cell growth; therefore, the selection of a proper agitation speed is required for the high production of 2,3-BD. Because the highest titer of 2,3-BD production using *R*. *ornithinolytica* B6 was achieved at 400 rpm, all further fed-batch fermentations were done at an agitation speed of 400 rpm.

### Enhancement of 2,3-BD production by overexpression of the *budABC* genes

In the fed-batch fermentation with *R*. *ornithinolytica* B6 (Table 3), although much less NADH-requiring metabolites (e.g., lactic acid, succinic acid, and ethanol) were produced at 400 rpm than at 300 rpm, acetic acid production at 400 rpm was significantly increased compared to that at 300 rpm. Because the partitioning of carbon flux at the pyruvate node to acetic acid (via acetyl Co-A) and 2,3-BD (via α-acetolactate) occurs, the 2,3-BD synthesis-related genes, acetolactate synthase (encoded by *budB*), acetolactate decarboxylase (encoded by *budA*), and AR (encoded by *budC*), were overexpressed to increase the carbon flux to 2,3-BD and consequently to decrease the carbon flux to acetic acid. The overexpression of *budABC* in *R*. *ornithinolytica* B6 (pBbA5c-*budABC*) was confirmed by SDS-PAGE as shown in S1 Fig.

**Fig 4:**
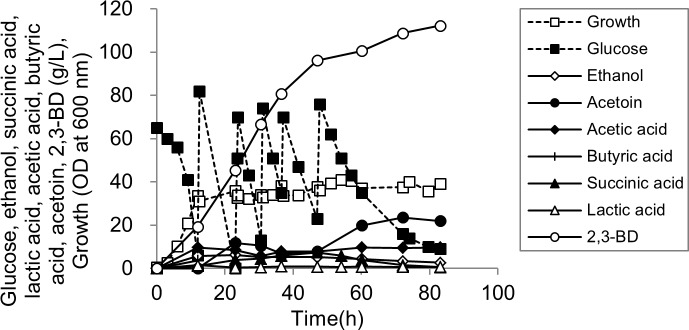
Fed-batch fermentation of glucose by *R*. *ornithinolytica* B6 (pBbA5c-*budABC*); ■, glucose; □, growth; ♢, ethanol; ●, acetoin; ○, 2,3-BD; ♦, acetic acid; ▲, succinic acid; △, lactic acid; +, butyric acid.

Finally, when *R*. *ornithinolytica* B6 harboring pBbA5c-*budABC* was used in the fed-batch fermentation at 400 rpm, 2,3-BD production was dramatically increased to 112.19 g/L (Fig 4) with a yield of 0.38 g/g and a productivity of 1.35 g/L/h which were higher than those of *R*. *ornithinolytica* B6 (pBbA5c) (i.e., the control) by 1.84-fold, 1.23-fold, and 1.88-fold, respectively (Table 3). Acetic acid production by *R*. *ornithinolytica* B6 (pBbA5c-*budABC*) was significantly reduced to 9.71 g/L compared to that of the control, indicating a decrease of the carbon flux at the pyruvate node to acetic acid. In contrast, *R*. *ornithinolytica* B6 harboring pBbA5c-*budABC* produced more acetoin than that of the control indicating that acetoin conversion to 2,3-BD was somewhat limited. Among the 2,3-BD synthesis-related genes, AR is known to catalyze both the reduction of acetoin to 2,3-BD and the reverse reaction (the oxidation of 2,3-BD to acetoin) [29,30]. Therefore, the activity of AR toward 2,3-BD synthesis was compared to that of the reverse reaction. When the crude protein extract from *R*. *ornithinolytica* B6 (pUC18CM-*budC*) was used to measure the AR activity, the AR activity toward acetoin reduction to 2,3-BD was 10-fold higher than that of the reverse activity (3.21 vs. 0.32 U/mg protein), implying that the oxidation of 2,3-BD to acetoin by AR would not be the reason for a high residual acetoin concentration.

## Discussion

This study is the first report on the high production of 2,3-BD using the wild type as well as the genetically engineered *Raoultella* species. *R*. *ornithinolytica* (formerly classified as *K*. *ornithinolytica*) is generally found in aquatic environmental samples, insects, and fishes. Unlike other *Klebsiella* species, human infections by *R*. *ornithinolytica* are very rare [19]. In fact, *R*. *ornithinolytica* B6 did not grow at 37°C (close to normal body temperature) which could be a reason for rare infections in humans by *R*. *ornithinolytica*. Recently, *R*. *ornithinolytica* has been reported as a possible symbiotic microorganism for earthworms, and it produces metabolites exhibiting antitumor, antifungal, and anticancer effects [21,22]. *R*. *ornithinolytica* was also shown to promote the degradation of microalgal cell wall through cellulolytic activities at low temperatures which resulted in an increase of biogas production [31]. In addition to those previous results, 2,3-BD production by *R*. *ornithinolytica* B6 can be a new application of *Raoultella* species. Furthermore, *R*. *ornithinolytica* B6 was able to utilize various sugars including glucose, galactose, fructose, xylose and sucrose. Because utilization of renewable lignocellulosic biomass containing various sugars has been attracting much attention, the broad substrate spectrum of *R*. *ornithinolytica* B6 is advantageous in utilizing lignocellulosic biomasses for 2,3-BD production.

Two operation strategies were used for the improvement of 2,3-BD production. One was the two stage pH control scheme. The initial pH was set at pH 7.0 for cell mass production in the early stage of fermentation, and then, pH was maintained at pH 5.5 after pH naturally reached 5.5 during the fermentation. In the case of the two pH control stage process, *R*. *ornithinolytica* B6 produced a higher 2,3-BD yield than that of the other pH control schemes (no pH control and pH control at 7). The other strategy was the optimization of the agitation speed which has been reported to be critical for high 2,3-BD production [2,9,13]. We conducted fed-batch fermentations at different agitation speeds to find the optimum oxygen supply for 2,3-BD production by *R*. *ornithinolytica* B6. As a result, the increased oxygen supply by controlling the agitation speed at 400 rpm improved not only the 2,3-BD concentration and productivity but also the 2,3-BD yield. This result is an advantageous and distinguished 2,3-BD production characteristic of *R*. *ornithinolytica* compared to previous reports with other 2,3-BD producers: a high oxygen supply resulted in a decrease in the 2,3-BD yield despite the increase in the 2,3-BD titer, productivity, and cell growth [2,9,13].

After optimizing the operation conditions, a genetically engineered *R*. *ornithinolytica* B6 strain was constructed to improve 2,3-BD production further. We overexpressed the *budABC* genes which are involved in the conversion of pyruvate to 2,3-BD. The enhancement of 2,3-BD production as well as a decrease in acetic acid formation by *R*. *ornithinolytica* B6 (pBbA5c-*budABC*) was remarkable compared to the results of the wild type (Table 3), showing a substantial increase of carbon flux to the 2,3-BD synthesis pathway at the pyruvate node. Consequently, the yield of 2,3-BD by *R*. *ornithinolytica* B6 (pBbA5c-*budABC*) was significantly improved compared to the yield with the control strain, *R*. *ornithinolytica* B6 (pBbA5c) (0.38 vs. 0.31 g/g). Unexpectedly, acetoin accumulated up to 22.02 g/L. In previous reports, high 2,3-BD production along with lower acetoin accumulation was attempted using a two-stage agitation speed control strategy in which the agitation speed was changed from a high rpm to a lower rpm [2,9]. When we applied a two-agitation speed strategy for *R*. *ornithinolytica* B6 (pBbA5c-*budABC*) fermentation by changing the agitation speed from 400 rpm to 300 rpm after 50 hours of fermentation, the acetoin concentration was reduced to 9.12 g/L; however, the 2,3-BD titer and productivity were also reduced to 101.13 g/L and 1.26 g/L/h, respectively. Future studies might be required for the increase of NADH availability or AR activity to reduce acetoin accumulation.

In this study, we investigated the high production of 2,3-BD with *R*. *ornithinolytica* B6 through the optimization of the fermentation conditions and genetic engineering. *R*. *ornithinolytica* B6 (pBbA5c-*budABC*) produced 2,3-BD up to 112.19 g/L by increasing the carbon flux to 2,3-BD. This is the first report to present a high 2,3-BD production by *R*. *ornithinolytica* strains, extending a microbial spectrum of 2,3-BD producer over previously known 2,3-BD producers. Comparing this result with previously reported 2,3-BD productions by *K*. *oxytoca* (95.5~ 142.5 g/L) [2,9–11,13], *K*. *pneumoniae* (90~ 150 g/L) [5,12,14], *S*. *marcescens* (87.8~ 152 g/L) [6,7,32], and *E*. *aerogenes* (118.05 g/L) [8], *R*. *ornithinolytica* B6 can be considered as a potential candidate for a high 2,3-BD production with expectation of enhanced 2,3-BD production through further metabolic engineering and medium optimization.

## Supporting Information

S1 Fig SDS-PAGE analysis of gene overexpression in the *R*.*ornithinolytica* B6 (pBbA5c-*budABC*) comparing with wild-type strain. The *rfp* (28 kDa), *budA*(29 kDa), *budB* (61 kDa) and *budC* (26 kDa) bands are indicated by arrows. The samples were prepared after 3 hours of induction with 0.5 mM IPTG (lanes 2,3,4); lane 1, *R*.*ornithinolytica* B6; lane 2, *R*.*ornithinolytica* B6 (pBbA5c); lane 3, *R*.*ornithinolytica* B6 (pBbA5c-RFP); lane 4, *R*.*ornithinolytica* B6 (pBbA5c-*budABC*).
(TIF)
