# Supplementary figures and images for: High Production of 2,3-Butanediol (2,3-BD) by Raoultella ornithinolytica B6 via Optimizing Fermentation Conditions and Overexpressing 2,3-BD Synthesis Genes
Source: PLoS One. 2016 Oct 19;11(10):e0165076. doi: 10.1371/journal.pone.0165076 (PMC5070830; doi:10.1371/journal.pone.0165076)

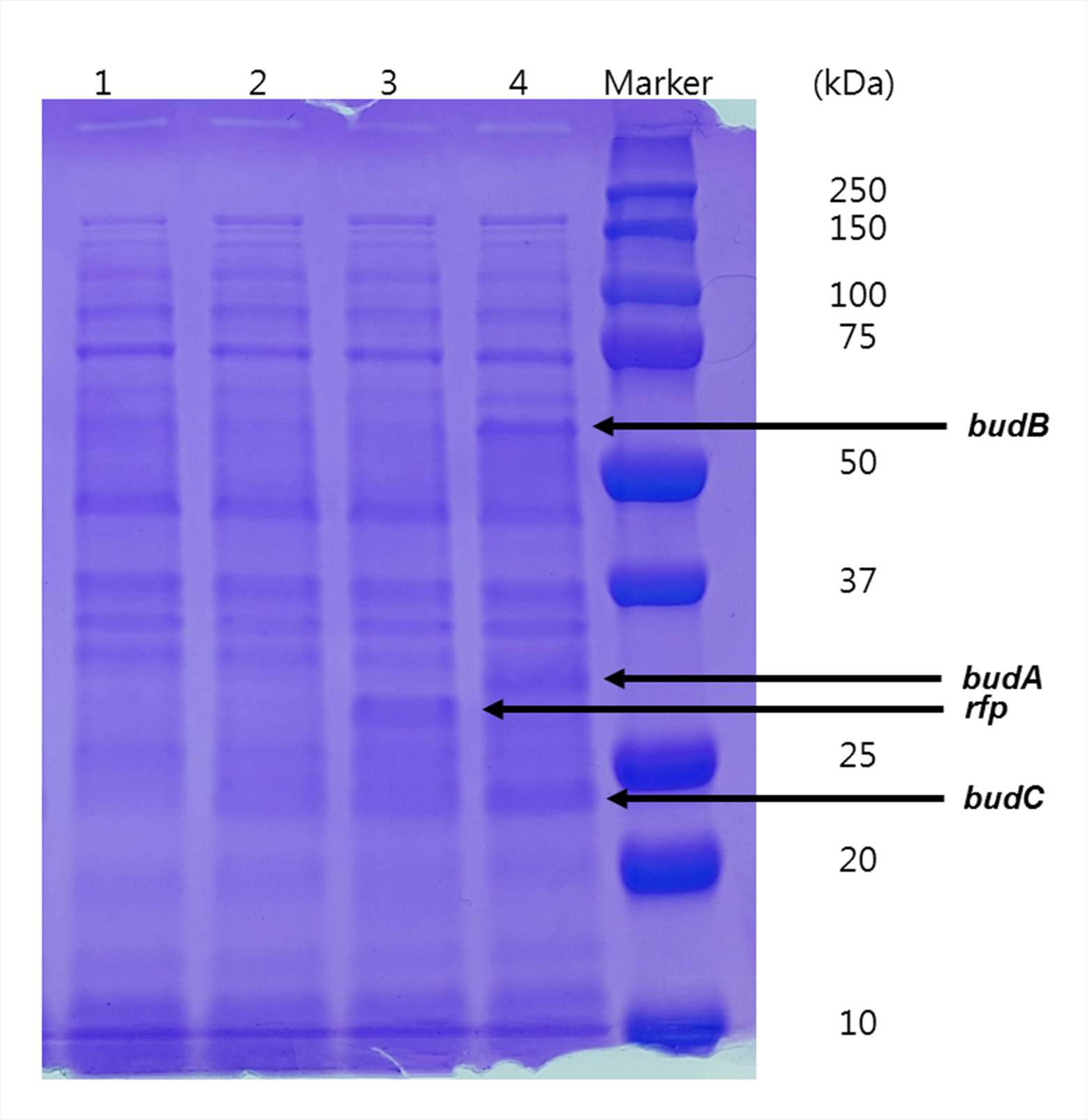

Supplement: S1 Fig — The rfp (28 kDa), budA(29 kDa), budB (61 kDa) and budC (26 kDa) bands are indicated by arrows. The samples were prepared after 3 hours of induction with 0.5 mM IPTG (lanes 2,3,4); lane 1, R.ornithinolytica B6; lane 2, R.ornithinolytica B6 (pBbA5c); lane 3, R.ornithinolytica B6 (pBbA5c-RFP); lane 4, R.ornithinolytica B6 (pBbA5c-budABC). (TIF) [file pone.0165076.s001.tif]
